# Bis[1-(2,6-dichloro­benz­yl)-3-methyl­pyrazin-1-ium] bis­(maleonitrile­dithiol­ato)nickelate(II)

**DOI:** 10.1107/S1600536808034776

**Published:** 2008-10-31

**Authors:** Shan-Shan Yu, Hua Xian, Zheng-Fang Tian

**Affiliations:** a Department of Chemistry, Nanjing Xiaozhuang College, Nanjing 210017, People’s Republic of China; b College of Chemistry and Applied Chemistry, Huanggang Normal University, Huanggang 438000, People’s Republic of China

## Abstract

In the crystal structure of the title compound, (C_12_H_11_Cl_2_N_2_)_2_[Ni(C_4_N_2_S_2_)_2_], the Ni^II^ complex dianion is located on an inversion centre. The Ni^II^ atom is coordinated by four S atoms in a square-planar geometry. In the cation, the dihedral angle between the benzene and pyrazine rings is 85.2 (2)°.

## Related literature

For general background, see: Ni *et al.* (2005 ▶); Nishijo *et al.* (2000 ▶); Robertson & Cronin (2002 ▶). For related structures, see: Ni *et al.* (2004 ▶); Ren *et al.* (2004 ▶).
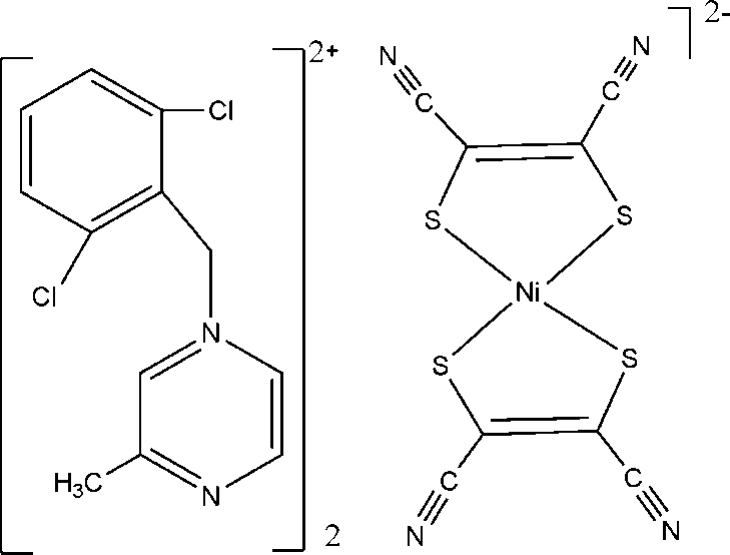


## Experimental

### 

#### Crystal data

(C_12_H_11_Cl_2_N_2_)_2_[Ni(C_4_N_2_S_2_)_2_]
*M* *_r_* = 847.33
Monoclinic, 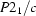

*a* = 9.081 (2) Å
*b* = 20.238 (5) Å
*c* = 10.489 (2) Å
β = 111.243 (4)°
*V* = 1796.6 (7) Å^3^
*Z* = 2
Mo *K*α radiation
μ = 1.11 mm^−1^
*T* = 298 (2) K
0.30 × 0.20 × 0.20 mm

#### Data collection

Bruker SMART CCD area-detector diffractometer
Absorption correction: multi-scan (**SADABS**; Bruker, 2000 ▶) *T*_min_ = 0.732, *T*_max_ = 0.809
8822 measured reflections
3159 independent reflections
2170 reflections with *I* > 2σ(*I*)
*R*_int_ = 0.086

#### Refinement

*R*[*F* ^2^ > 2σ(*F* ^2^)] = 0.055
*wR*(*F* ^2^) = 0.114
*S* = 0.96
3159 reflections
224 parameters
H-atom parameters constrained
Δρ_max_ = 0.51 e Å^−3^
Δρ_min_ = −0.30 e Å^−3^

### 

Data collection: *SMART* (Bruker, 2000 ▶); cell refinement: *SAINT* (Bruker, 2000 ▶); data reduction: *SAINT*; program(s) used to solve structure: *SHELXS97* (Sheldrick, 2008 ▶); program(s) used to refine structure: *SHELXL97* (Sheldrick, 2008 ▶); molecular graphics: *SHELXTL* (Sheldrick, 2008 ▶); software used to prepare material for publication: *SHELXTL*.

## Supplementary Material

Crystal structure: contains datablocks global, I. DOI: 10.1107/S1600536808034776/is2350sup1.cif

Structure factors: contains datablocks I. DOI: 10.1107/S1600536808034776/is2350Isup2.hkl

Additional supplementary materials:  crystallographic information; 3D view; checkCIF report

## Figures and Tables

**Fig. 1.**
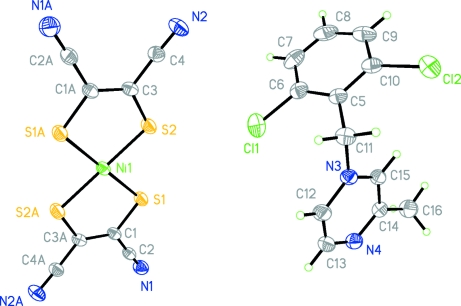
The molecular structure of (I), showing the atom-numbering scheme and displacement ellipsoids at the 30% probability level. The suffix A corresponds to symmetry code (-x, -y+1, -z).

## supplementary crystallographic information

### Comment

Molecular solids based on transition metal dithiolene complexes have attracted intense interest in recent years, not only owing to the fundamental research of magnetic interactions and magneto-structural correlations but also to the development of new functional molecule-based materials (Robertson & Cronin, 2002). Much work has been performed in molecular solids based on *M*[dithiolene]_2_ complexes owing to their application as building blocks in molecular-based materials showing magnetic, superconducting and optical properties (Nishijo *et al.*, 2000; Ni *et al.*, 2005). Herein, we report the crystal structure of the title compound, (I).

The molecular structure of (I) is illustrated in Fig. 1. Compound (I) crystallizes in monoclinic system, with one half [Ni(mnt)_2_]^2-^ dianion and one 1-(2,6-dichlorobenzyl)-3-methylpyrazine cation in an asymmetric unit. The anion [Ni(mnt)_2_]^2-^ possesses an approximated planar geometry and most of the bond lengths and angles are in good agreement with the various [Ni(mnt)_2_]^2-^ compounds (Ni *et al.*, 2004; Ren *et al.*, 2004).

### Experimental

Disodium maleonitriledithiolate (456 mg, 2.5 mmol) and nickel chloride hexahydrate (297 mg, 1.25 mmol) were mixed under stirring in water (20 mL) at room temperature. Subsequently, a solution of 1-(2,6-dichlorobenzyl)-3-methylpyrazine iodide (952 mg, 2.5 mmol) in methanol (10 mL) was added to the mixture. The black precipitate that was immediately formed was filtered off and washed with methanol. The crude product was recrystallized in acetone (20 mL) to give black block crystals. Anal. Calcd. for C_32_H_22_Cl_4_N_8_NiS_4_: C 48.73, H 2.81, N 14.21%. Found: C 48.69, H 2.78, N 14.09%.

### Refinement

The H atoms were placed in geometrically idealized positions (C—H = 0.93–0.97 Å) and refined as riding atoms, with *U*_iso_(H) = 1.2*U*_eq_(C).

### Crystal data

**Table:**

(C_12_H_11_Cl_2_N_2_)_2_[Ni(C_4_N_2_S_2_)_2_]	*F*(000) = 860
*M**_r_* = 847.33	*D*_x_ = 1.566 Mg m^−^^3^
Monoclinic, *P*2_1_/*c*	Mo *K*α radiation, λ = 0.71073 Å
Hall symbol: -P 2ybc	Cell parameters from 773 reflections
*a* = 9.081 (2) Å	θ = 2.6–21.2°
*b* = 20.238 (5) Å	µ = 1.11 mm^−^^1^
*c* = 10.489 (2) Å	*T* = 298 K
β = 111.243 (4)°	Block, black
*V* = 1796.6 (7) Å^3^	0.30 × 0.20 × 0.20 mm
*Z* = 2	

### Data collection

**Table:**

Bruker SMART CCD area-detector diffractometer	3159 independent reflections
Radiation source: fine-focus sealed tube	2170 reflections with *I* > 2σ(*I*)
graphite	*R*_int_ = 0.086
φ and ω scans	θ_max_ = 25.0°, θ_min_ = 2.0°
Absorption correction: multi-scan (*SADABS*; Bruker, 2000)	*h* = −10→6
*T*_min_ = 0.732, *T*_max_ = 0.809	*k* = −24→23
8822 measured reflections	*l* = −12→12

### Refinement

**Table:**

Refinement on *F*^2^	Primary atom site location: structure-invariant direct methods
Least-squares matrix: full	Secondary atom site location: difference Fourier map
*R*[*F*^2^ > 2σ(*F*^2^)] = 0.055	Hydrogen site location: inferred from neighbouring sites
*wR*(*F*^2^) = 0.114	H-atom parameters constrained
*S* = 0.96	*w* = 1/[σ^2^(*F*_o_^2^) + (0.0362*P*)^2^] where *P* = (*F*_o_^2^ + 2*F*_c_^2^)/3
3159 reflections	(Δ/σ)_max_ = 0.001
224 parameters	Δρ_max_ = 0.51 e Å^−^^3^
0 restraints	Δρ_min_ = −0.30 e Å^−^^3^

### Special details

**Table:**

Geometry. All e.s.d.'s (except the e.s.d. in the dihedral angle between two l.s. planes) are estimated using the full covariance matrix. The cell e.s.d.'s are taken into account individually in the estimation of e.s.d.'s in distances, angles and torsion angles; correlations between e.s.d.'s in cell parameters are only used when they are defined by crystal symmetry. An approximate (isotropic) treatment of cell e.s.d.'s is used for estimating e.s.d.'s involving l.s. planes.
Refinement. Refinement of *F*^2^ against ALL reflections. The weighted *R*-factor *wR* and goodness of fit *S* are based on *F*^2^, conventional *R*-factors *R* are based on *F*, with *F* set to zero for negative *F*^2^. The threshold expression of *F*^2^ > σ(*F*^2^) is used only for calculating *R*-factors(gt) *etc*. and is not relevant to the choice of reflections for refinement. *R*-factors based on *F*^2^ are statistically about twice as large as those based on *F*, and *R*- factors based on ALL data will be even larger.

### Fractional atomic coordinates and isotropic or equivalent isotropic displacement parameters (Å^2^)

**Table:**

	*x*	*y*	*z*	*U*_iso_*/*U*_eq_	
Ni1	0.0000	0.5000	0.0000	0.0410 (2)	
C1	0.1965 (5)	0.45625 (18)	−0.1702 (4)	0.0434 (10)	
C2	0.3109 (6)	0.45822 (19)	−0.2353 (5)	0.0475 (11)	
C3	−0.0857 (5)	0.59184 (19)	0.1944 (4)	0.0445 (11)	
C4	−0.0777 (5)	0.6476 (2)	0.2820 (5)	0.0489 (11)	
C5	0.5497 (5)	0.67689 (19)	0.7483 (4)	0.0418 (10)	
C6	0.4392 (5)	0.6850 (2)	0.6168 (5)	0.0508 (11)	
C7	0.3690 (6)	0.7445 (3)	0.5682 (5)	0.0676 (14)	
H7	0.2936	0.7477	0.4804	0.081*	
C8	0.4110 (6)	0.7986 (2)	0.6501 (6)	0.0735 (16)	
H8	0.3661	0.8394	0.6172	0.088*	
C9	0.5185 (6)	0.7936 (2)	0.7801 (6)	0.0675 (14)	
H9	0.5465	0.8309	0.8356	0.081*	
C10	0.5857 (5)	0.7331 (2)	0.8293 (5)	0.0505 (12)	
C11	0.6274 (5)	0.61171 (19)	0.7983 (4)	0.0486 (11)	
H11A	0.6794	0.6130	0.8970	0.058*	
H11B	0.5479	0.5772	0.7759	0.058*	
C12	0.7394 (5)	0.53854 (19)	0.6677 (5)	0.0512 (12)	
H12	0.6629	0.5071	0.6629	0.061*	
C13	0.8486 (6)	0.5274 (2)	0.6073 (4)	0.0524 (12)	
H13	0.8429	0.4881	0.5597	0.063*	
C14	0.9720 (5)	0.6256 (2)	0.6865 (4)	0.0448 (10)	
C15	0.8622 (5)	0.63866 (18)	0.7459 (4)	0.0408 (10)	
H15	0.8696	0.6776	0.7949	0.049*	
C16	1.1035 (5)	0.6723 (2)	0.7000 (5)	0.0666 (14)	
H16A	1.2027	0.6502	0.7430	0.100*	
H16B	1.0971	0.7094	0.7547	0.100*	
H16C	1.0953	0.6874	0.6109	0.100*	
Cl1	0.38504 (16)	0.61665 (7)	0.50983 (14)	0.0775 (4)	
Cl2	0.71852 (16)	0.72925 (6)	0.99706 (13)	0.0719 (4)	
N1	0.4021 (5)	0.46159 (18)	−0.2871 (4)	0.0651 (12)	
N2	−0.0664 (5)	0.69443 (19)	0.3446 (4)	0.0688 (12)	
N3	0.7460 (4)	0.59632 (15)	0.7340 (3)	0.0415 (9)	
N4	0.9619 (4)	0.57012 (18)	0.6139 (4)	0.0535 (10)	
S1	0.20391 (14)	0.51963 (5)	−0.05585 (12)	0.0484 (3)	
S2	0.05266 (14)	0.59091 (5)	0.11563 (12)	0.0532 (3)	

### Atomic displacement parameters (Å^2^)

**Table:**

	*U*^11^	*U*^22^	*U*^33^	*U*^12^	*U*^13^	*U*^23^
Ni1	0.0481 (5)	0.0298 (4)	0.0442 (5)	0.0004 (3)	0.0155 (4)	−0.0004 (3)
C1	0.053 (3)	0.030 (2)	0.048 (3)	0.002 (2)	0.018 (2)	0.0027 (18)
C2	0.058 (3)	0.031 (2)	0.054 (3)	−0.002 (2)	0.021 (3)	0.000 (2)
C3	0.054 (3)	0.034 (2)	0.047 (3)	0.005 (2)	0.020 (2)	−0.0004 (19)
C4	0.056 (3)	0.037 (3)	0.056 (3)	0.001 (2)	0.024 (2)	0.002 (2)
C5	0.040 (3)	0.036 (2)	0.057 (3)	−0.0008 (18)	0.027 (2)	0.006 (2)
C6	0.042 (3)	0.050 (3)	0.066 (3)	−0.005 (2)	0.027 (2)	0.005 (2)
C7	0.049 (3)	0.075 (4)	0.077 (4)	0.015 (3)	0.021 (3)	0.031 (3)
C8	0.075 (4)	0.045 (3)	0.113 (5)	0.025 (3)	0.049 (4)	0.021 (3)
C9	0.079 (4)	0.044 (3)	0.098 (4)	0.008 (3)	0.053 (3)	0.001 (3)
C10	0.051 (3)	0.045 (3)	0.066 (3)	0.005 (2)	0.034 (2)	0.005 (2)
C11	0.055 (3)	0.043 (2)	0.057 (3)	−0.001 (2)	0.031 (2)	0.005 (2)
C12	0.052 (3)	0.033 (2)	0.066 (3)	0.000 (2)	0.019 (2)	0.003 (2)
C13	0.059 (3)	0.041 (3)	0.054 (3)	0.005 (2)	0.016 (2)	−0.004 (2)
C14	0.042 (3)	0.046 (3)	0.046 (3)	0.001 (2)	0.015 (2)	0.003 (2)
C15	0.044 (3)	0.029 (2)	0.047 (3)	−0.0017 (19)	0.014 (2)	0.0026 (18)
C16	0.056 (3)	0.059 (3)	0.089 (4)	−0.011 (2)	0.032 (3)	−0.007 (3)
Cl1	0.0753 (10)	0.0753 (9)	0.0747 (10)	−0.0217 (7)	0.0185 (7)	−0.0094 (7)
Cl2	0.0806 (10)	0.0729 (9)	0.0635 (9)	−0.0026 (7)	0.0276 (7)	−0.0100 (6)
N1	0.070 (3)	0.052 (3)	0.083 (3)	−0.004 (2)	0.040 (3)	−0.001 (2)
N2	0.091 (3)	0.045 (2)	0.076 (3)	−0.004 (2)	0.036 (2)	−0.017 (2)
N3	0.046 (2)	0.0282 (19)	0.049 (2)	0.0030 (15)	0.0164 (17)	0.0058 (15)
N4	0.053 (3)	0.054 (2)	0.053 (2)	0.0067 (19)	0.0196 (19)	−0.0017 (19)
S1	0.0551 (8)	0.0365 (6)	0.0549 (8)	−0.0057 (5)	0.0214 (6)	−0.0049 (5)
S2	0.0621 (8)	0.0388 (6)	0.0658 (8)	−0.0115 (5)	0.0316 (6)	−0.0107 (5)

### Geometric parameters (Å, °)

**Table:**

Ni1—S2	2.1596 (11)	C8—H8	0.9300
Ni1—S2^i^	2.1596 (11)	C9—C10	1.382 (6)
Ni1—S1^i^	2.1715 (12)	C9—H9	0.9300
Ni1—S1	2.1715 (12)	C10—Cl2	1.737 (5)
C1—C3^i^	1.356 (5)	C11—N3	1.496 (5)
C1—C2	1.436 (6)	C11—H11A	0.9700
C1—S1	1.741 (4)	C11—H11B	0.9700
C2—N1	1.145 (5)	C12—N3	1.351 (5)
C3—C1^i^	1.356 (5)	C12—C13	1.375 (6)
C3—C4	1.441 (6)	C12—H12	0.9300
C3—S2	1.736 (4)	C13—N4	1.326 (5)
C4—N2	1.136 (5)	C13—H13	0.9300
C5—C10	1.387 (5)	C14—N4	1.341 (5)
C5—C6	1.391 (6)	C14—C15	1.379 (5)
C5—C11	1.498 (5)	C14—C16	1.489 (6)
C6—C7	1.370 (6)	C15—N3	1.330 (5)
C6—Cl1	1.737 (4)	C15—H15	0.9300
C7—C8	1.358 (7)	C16—H16A	0.9600
C7—H7	0.9300	C16—H16B	0.9600
C8—C9	1.363 (7)	C16—H16C	0.9600
			
S2—Ni1—S2^i^	180.00 (3)	C5—C10—Cl2	120.4 (3)
S2—Ni1—S1^i^	92.37 (4)	N3—C11—C5	110.5 (3)
S2^i^—Ni1—S1^i^	87.63 (4)	N3—C11—H11A	109.6
S2—Ni1—S1	87.63 (4)	C5—C11—H11A	109.6
S2^i^—Ni1—S1	92.37 (4)	N3—C11—H11B	109.6
S1^i^—Ni1—S1	180.0	C5—C11—H11B	109.6
C3^i^—C1—C2	123.1 (4)	H11A—C11—H11B	108.1
C3^i^—C1—S1	119.9 (3)	N3—C12—C13	118.3 (4)
C2—C1—S1	117.0 (3)	N3—C12—H12	120.8
N1—C2—C1	178.2 (4)	C13—C12—H12	120.8
C1^i^—C3—C4	123.0 (4)	N4—C13—C12	122.9 (4)
C1^i^—C3—S2	121.3 (3)	N4—C13—H13	118.5
C4—C3—S2	115.7 (3)	C12—C13—H13	118.5
N2—C4—C3	174.5 (5)	N4—C14—C15	120.4 (4)
C10—C5—C6	115.8 (4)	N4—C14—C16	118.2 (4)
C10—C5—C11	122.1 (4)	C15—C14—C16	121.4 (4)
C6—C5—C11	122.1 (4)	N3—C15—C14	120.9 (4)
C7—C6—C5	123.0 (4)	N3—C15—H15	119.6
C7—C6—Cl1	118.3 (4)	C14—C15—H15	119.6
C5—C6—Cl1	118.7 (3)	C14—C16—H16A	109.5
C8—C7—C6	119.1 (5)	C14—C16—H16B	109.5
C8—C7—H7	120.5	H16A—C16—H16B	109.5
C6—C7—H7	120.5	C14—C16—H16C	109.5
C7—C8—C9	120.6 (5)	H16A—C16—H16C	109.5
C7—C8—H8	119.7	H16B—C16—H16C	109.5
C9—C8—H8	119.7	C15—N3—C12	119.4 (4)
C8—C9—C10	119.9 (5)	C15—N3—C11	120.0 (3)
C8—C9—H9	120.0	C12—N3—C11	120.5 (3)
C10—C9—H9	120.0	C13—N4—C14	117.9 (4)
C9—C10—C5	121.6 (4)	C1—S1—Ni1	103.14 (15)
C9—C10—Cl2	118.0 (4)	C3—S2—Ni1	103.02 (14)
			
C10—C5—C6—C7	0.1 (6)	C16—C14—C15—N3	−179.5 (4)
C11—C5—C6—C7	−178.7 (4)	C14—C15—N3—C12	2.8 (6)
C10—C5—C6—Cl1	−178.9 (3)	C14—C15—N3—C11	−179.5 (4)
C11—C5—C6—Cl1	2.3 (5)	C13—C12—N3—C15	−3.9 (6)
C5—C6—C7—C8	1.6 (7)	C13—C12—N3—C11	178.4 (4)
Cl1—C6—C7—C8	−179.4 (4)	C5—C11—N3—C15	56.1 (5)
C6—C7—C8—C9	−1.7 (8)	C5—C11—N3—C12	−126.2 (4)
C7—C8—C9—C10	0.1 (8)	C12—C13—N4—C14	2.1 (6)
C8—C9—C10—C5	1.7 (7)	C15—C14—N4—C13	−3.3 (6)
C8—C9—C10—Cl2	−178.5 (4)	C16—C14—N4—C13	177.1 (4)
C6—C5—C10—C9	−1.7 (6)	C3^i^—C1—S1—Ni1	−4.5 (4)
C11—C5—C10—C9	177.1 (4)	C2—C1—S1—Ni1	175.2 (3)
C6—C5—C10—Cl2	178.5 (3)	S2—Ni1—S1—C1	−175.14 (14)
C11—C5—C10—Cl2	−2.7 (5)	S2^i^—Ni1—S1—C1	4.86 (14)
C10—C5—C11—N3	−105.5 (4)	C1^i^—C3—S2—Ni1	−2.9 (4)
C6—C5—C11—N3	73.2 (5)	C4—C3—S2—Ni1	179.2 (3)
N3—C12—C13—N4	1.5 (6)	S1^i^—Ni1—S2—C3	4.39 (15)
N4—C14—C15—N3	0.9 (6)	S1—Ni1—S2—C3	−175.61 (15)

Symmetry codes: (i) −*x*, −*y*+1, −*z*.
